# The “Endothelialized Muscularis Mucosae”: A Case Report Describing a Large Cavernous Hemangioma at the Terminal Ileum and a New Histologic Clue for Preoperative Diagnosis from Endoscopic Biopsy

**DOI:** 10.1155/2015/454836

**Published:** 2015-09-09

**Authors:** Erin K. Purdy-Payne, Jean F. Miner, Brandon Foles, Tien-Anh N. Tran

**Affiliations:** ^1^University of Central Florida College of Medicine, 6850 Lake Nona Boulevard, Orlando, FL 32827, USA; ^2^Department of Surgery, Florida Hospital Orlando, 2415 North Orange Avenue, Suite 400, Orlando, FL 32803, USA; ^3^Department of Pathology, Florida Hospital Orlando, 601 E. Rollins Street, Orlando, FL 32803, USA

## Abstract

Cavernous hemangiomas of the gastrointestinal tract are quite rare and, until now, have been difficult to diagnose preoperatively due their nonspecific presentations and imaging features, as well as a lack of histologic description pertaining to small superficial biopsies such as those obtained endoscopically. We report a unique case of a 4 cm transmural cavernous hemangioma in the terminal ileum with literature review and describe a new histologic finding—the “endothelialized muscularis mucosae,” which was discovered upon review of the endoscopic biopsy and could potentially facilitate preoperative diagnosis of these lesions from endoscopic biopsies in the future. These lesions have classically required surgical resection in order to make a definitive diagnosis and rule out malignancy, with which they share many historical and radiographic features. Due to their potential to cause bowel obstruction, intussusception, perforation, and hemorrhage, these lesions may ultimately require surgical resection to relieve symptoms or prevent or treat complications—however, surgical planning and patient counseling could be greatly improved by a preoperative diagnosis. Therefore, gastroenterologists, pathologists, and surgeons should be aware of the “endothelialized muscularis mucosae” which can be very helpful in diagnosing GI cavernous hemangiomas from endoscopic biopsies.

## 1. Introduction

Cavernous hemangiomas of the gastrointestinal tract are quite rare and, until now, difficult to diagnose preoperatively [1] due to a dearth of specific histologic description pertaining to small superficial biopsies such as those obtained endoscopically [2, 3]. We report a unique case of a 4 cm transmural cavernous hemangioma in the terminal ileum with literature review and describe a new histologic finding—the “endothelialized muscularis mucosae,” which was discovered upon review of the endoscopic biopsy and could potentially facilitate preoperative diagnosis of these lesions from endoscopic biopsies in the future.

## 2. Case Description

The patient was a 20-year-old female who presented to the emergency department with a progressive 2-week history of diffuse intermittent abdominal pain, which was aching at rest and sharp upon movement. It was accompanied by nausea for the past two days, with one episode of vomiting. She denied recent changes in her stool but reported passing stool only once per week for the past few years.

The patient also reported a 2-year history of vasovagal syncope, with an increase in presyncopal episodes during the 2-week course of her abdominal pain. These episodes were sometimes associated with heart palpitations, shortness of breath, and chest pain, which had previously been diagnosed as anxiety and gastroesophageal reflux disease. The history was otherwise noncontributory.

The patient's vital signs were unremarkable and she was afebrile. There were no masses or tenderness upon abdominal palpation. Initial labs were within normal limits, and screening tests were negative for pregnancy and sexually transmitted infections.

**Figure 1:**
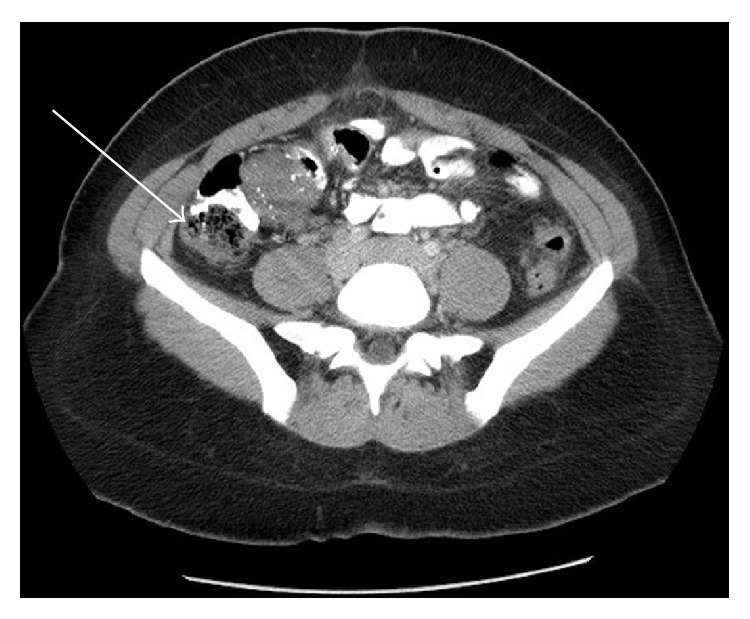
Computed tomography without contrast of the abdomen and pelvis was notable for a 4.7 cm mass involving the wall of the terminal ileum, with speckled calcifications, adjacent lymphadenopathy, and a small amount of dense fluid in the pelvis (CT, with arrow).

**Figure 2:**
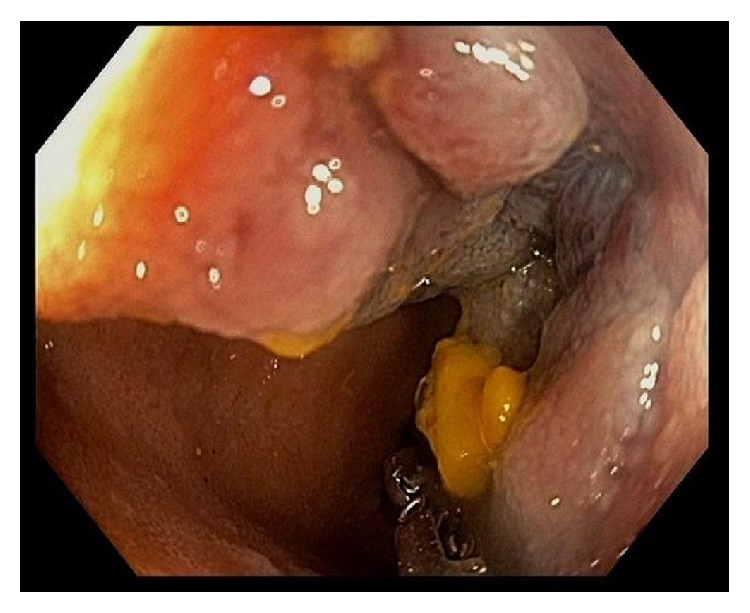
On colonoscopy, the luminal surface of the mass was visible through the ileocecal valve and was described as a 4-5 cm friable mass in the terminal ileum (endoscopic view).

A computed tomography of the abdomen and pelvis without contrast was notable for a 4.7 cm mass involving the wall of the terminal ileum, with speckled calcifications, adjacent lymphadenopathy, and a small amount of dense fluid in the pelvis (Figure 1). No hepatic abnormalities were visualized. Colonoscopy demonstrated a 4-5 cm friable mass in the terminal ileum (Figure 2), and multiple biopsies of this area were taken. The biopsies were originally described as unremarkable small bowel mucosa with preserved villous architecture.

**Figure 3:**
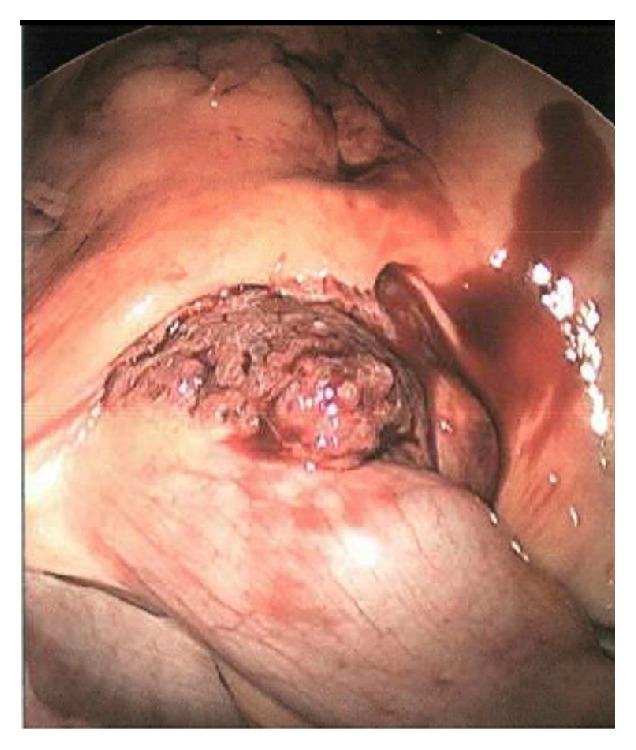
Laparoscopy revealed a soft, maroon, cystic mass. It was noted to be friable with scant bleeding and to have a tortuous, dilated blood supply (laparoscopic view).

The patient subsequently underwent laparoscopic evaluation and resection of the mass. Upon entry into the abdomen, a small amount of blood was noted to be pooled in dependent locations within the abdomen and pelvis. At the terminal ileum, a soft, maroon, cystic, friable mass associated with scant bleeding and a tortuous, dilated blood supply was noted (Figure 3). A laparoscopic ileocecectomy was completed without any perioperative complications.

**Figure 4:**
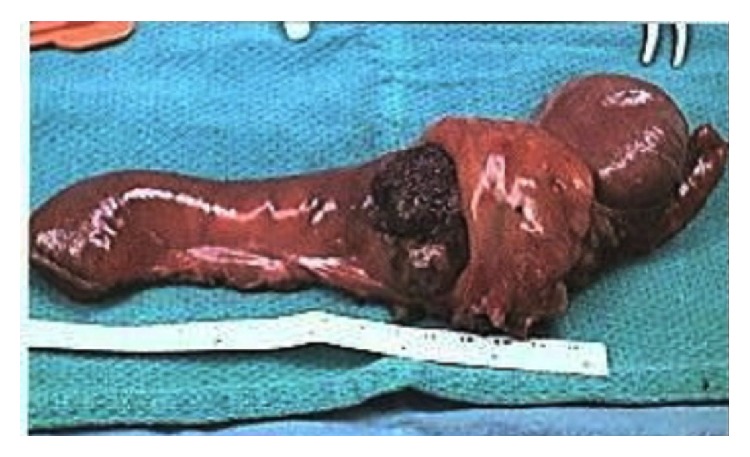
The gross resected specimen included an 11 cm segment of terminal ileum and 4 cm portion of cecum, with a 4 × 3.5 × 3 cm dark red, spongy mass at the terminal ileum (gross specimen).

**Figure 5:**
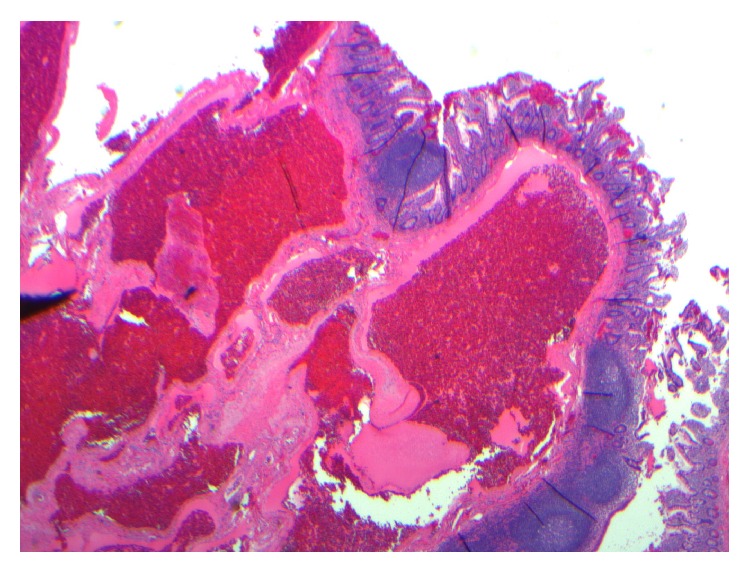
Histologic examination of the surgical specimen showed dilated vascular spaces filled with blood and lined by a layer of flat, benign-appearing endothelial cells. The wall of the vascular channels was composed of smooth muscle and fibrous tissue (histology of surgical specimen).

The resected specimen was composed of an 11 cm segment of terminal ileum and 4 cm portion of cecum (Figure 4). Gross examination revealed a 4 × 3.5 × 3 cm dark red, spongy mass at the terminal ileum that involved all layers of the small bowel and extended into the attached adipose tissue. Upon opening the bowel, a large amount of blood was noted within the lumen of the terminal ileum. The corresponding mucosal surface of the lesion was seeping fluid blood into the lumen. Histologic examination showed dilated vascular spaces, filled with blood and lined by a layer of flat, benign-appearing endothelial cells. The walls of the vascular channels were composed of smooth muscle and fibrous tissue (Figure 5). Calcifications and calcified phleboliths were observed in the wall and lumen of the vascular lesion. The diagnosis of a cavernous hemangioma was rendered.

**Figure 6:**
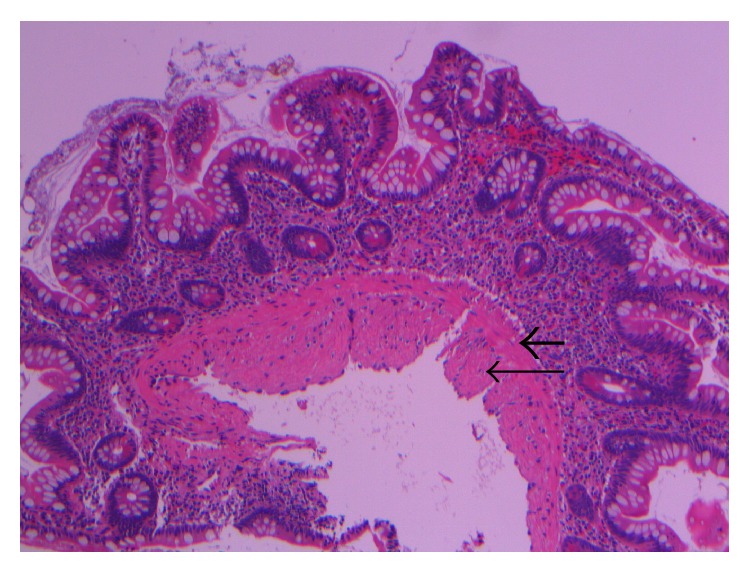
Histologic review of the original endoscopic biopsy revealed the so-called “endothelialized muscularis mucosae” characterized by a second layer of fibromuscular tissue (long, fine arrow) apposed to the muscularis mucosae (short, thick arrow) (histology of endoscopic biopsy, with arrows).

**Figure 7:**
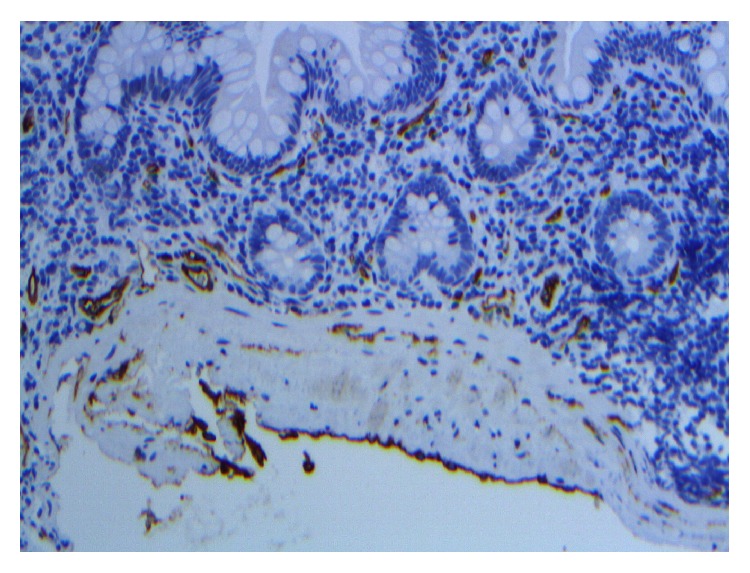
Upon immunohistochemical staining of the original endoscopic biopsy specimen, this second layer of smooth muscle/fibrous tissue was also lined on the antiluminal side by a layer of flat endothelial cells, highlighted by the endothelial marker CD34 (immunofluorescence staining of endoscopic biopsy).

Review of the original biopsy revealed a second layer of fibromuscular tissue apposed to the muscularis mucosae of the small bowel mucosa. On the antiluminal side of the intestinal lumen, this second layer of fibromuscular tissue was lined by a layer of flat endothelial cells (Figure 6). Immunohistochemical studies with the endothelial markers CD31 and CD34 highlighted the layer of endothelial cells lining one side of the second fibromuscular layer (Figure 7).

## 3. Discussion

Intestinal hemangiomas are very rare, with multiple histologic variations accounting for a combined incidence of only 0.05% of all gastrointestinal tract (GI) tumors [3]. They are more likely to be found in the jejunum [4], in contrast with most other GI tumors, only 3–6% of which reside in the small bowel [1]. A literature search for cavernous hemangiomas of the terminal ileum revealed only one prior case, which described a 5 cm cavernous hemangioma that was associated with perforation located near the origin of an ileocecal intussusception [3].

Small bowel tumors pose several diagnostic challenges, beginning with their nonspecific symptoms of abdominal pain, nausea, and distension, which could be more easily explained by many prevalent physiologic GI issues. Additionally, many are asymptomatic until later stages. Therefore they are often discovered either incidentally, or after a wide-ranging work-up and a significant delay since the onset of symptoms [1]. Late stage small bowel tumors may present suddenly, with acute intestinal obstruction or frank GI bleeding [1, 5–7], or insidiously, with subacute partial obstruction or chronic anemia secondary to occult GI bleeding [1, 8, 9]. Lesions involving the terminal ileum, such as in our case, may be more likely to present with obstructive symptoms, due to their impediment of this region's intestinal wall motility which is crucial to propulsion of contents through the ileocecal valve [10]. Our patient's dilated terminal ileum and pattern of passing stool only once per week suggest chronic subacute obstruction, likely due to impaired peristalsis at the terminal ileum. Her presenting symptoms of abdominal pain, nausea, and vomiting indicate progression towards a more acute obstruction.

Compared to other small bowel tumors, cavernous hemangiomas are more often symptomatic and more likely to present with anemia or bleeding, rarely even causing massive hemorrhaging [4, 6, 11]. With transmural lesions such as in our case, bleeding may occur not only intraluminally but also intra-abdominally [6]. In our patient, the blood noted in the abdominal cavity had likely been causing her sharp abdominal pain secondary to peritoneal irritation. Intestinal hemangiomas have also been associated with complications such as intussusception and perforation [3, 11], or platelet sequestration leading to thrombocytopenia [4]. In some cases, these complications can be life threatening.

Nonspecific features on imaging and limitations in visualization pose additional challenges to preoperative diagnosis of small bowel tumors. Patients who present with abdominal pain or obstructive symptoms are often evaluated by computed tomography (CT), which has a poor detection rate for small bowel tumors and is unlikely to visualize most cavernous hemangiomas [1, 12]. For lesions large enough to visualize, a CT scan can help determine the presence, size, and extension into nearby structures [7] but is usually unhelpful at differentiating the type of lesion [1]. A large cavernous hemangioma may be seen on CT scan as intestinal wall thickening which is persistently enhancing with IV contrast, or which may infiltrate nearby structures such as mesentery or abdominal wall. These features can be misleading and may cause a cavernous hemangioma to be mistaken for a more common tumor such as lymphoma [8]. CT scans can also demonstrate calcifications such as phleboliths, which occur due to vascular thrombosis within the tumor's dilated varicosities [4, 11, 12].

Patients who present with recurrent anemia or GI bleeding due to these lesions are often evaluated by traditional endoscopy, which is unable to reach most of the small bowel, or by capsule endoscopy [1]. When they are visualized on endoscopy, features of a cavernous hemangioma may include a purple or blue color, a nodular gross appearance, and hypervascularity, possibly with friability or visible bleeding [7, 8, 12]. Adjacent features may include mucosal congestion and submucosal dilated varicosities [3, 12]. Other diagnostic modalities, such as radionucleotide imaging or angiography showing hypervascularity, or barium studies demonstrating a nodular luminal defect which is compressible upon air insufflation, may help localize the lesion but cannot render a specific diagnosis [11, 12].

Little has been written in the literature on the histologic findings in biopsies of cavernous hemangiomas of the gastrointestinal tract. Accordingly, even lesions accessible for biopsy have evaded diagnosis due to inconclusive findings [2], and cavernous hemangiomas have almost exclusively required surgical resection for a definitive diagnosis [1, 12]. However, upon review of the original endoscopic biopsy in this case, we observed a second layer of fibromuscular tissue which was tightly apposed to the muscularis mucosae of the small bowel mucosa, with a layer of flat endothelial cells lining the antiluminal side of the second fibromuscular layer. Since duplication of the muscularis mucosae can occur in chronic diseases of the GI tract, immunohistochemical studies with the endothelial markers CD31 and CD34 were particularly helpful in highlighting the flat endothelial cells on the antiluminal side of the second fibromuscular layer. We therefore coined the term “endothelialized muscularis mucosae” to describe this phenomenon. In the future, this histologic sign could potentially aid preoperative diagnosis of GI tract cavernous hemangiomas by serving as a useful diagnostic clue for interpreting small biopsies such as those obtained during endoscopy. Preoperative histologic diagnosis would be helpful in counseling patients on the indications, risks, and benefits of surgery, as well as in planning the operation and the extent of the resection.

While cavernous hemangiomas are benign, they can be locally destructive by way of invading, exerting pressure, or disrupting the physiology of surrounding structures. Due to these factors and the prevalence of complications such as bleeding or bowel obstruction, cavernous hemangiomas require surgical resection more often than other intestinal hemangiomas [4]. Surgery is indicated for a variety of reasons, including to treat or prevent such complications, to relieve symptoms such as abdominal pain, or to rule out malignancy and acquire a diagnosis when the work-up has been nondiagnostic [4, 12]. In this case, all of these factors contributed to the decision for surgical resection, but we believe that surgical planning would have been greatly facilitated by a preoperative knowledge of the diagnosis.

Due to the nonspecific presentation, many patients with GI cavernous hemangiomas will be evaluated with endoscopy at some point, if not early on in their work-up [1]. When these lesions are accessible by traditional endoscopy, they present a problem for the patient and the provider, if the lesion is detected endoscopically but is unable to be diagnosed histologically. The first issue is that an unknown lesion may or may not require resection depending on its histologic diagnosis, but resection is necessary to acquire the histologic diagnosis in the first place. Furthermore, a surgeon in this position must debate whether to perform an excisional biopsy only, subjecting the patient to another abdominal surgery if the histologic diagnosis is malignant, or whether to initially resect a larger portion of the bowel and mesentery, in hopes that the patient will require only one operation to cover the anticipated histologic diagnostic possibilities. At this point in the work-up, either operation may be unnecessary to manage the lesion, depending on the patient's symptoms and the likelihood of complications. For these reasons, we believe our new histologic observation of the “endothelialized muscularis mucosae” has an important role in identifying these cavernous hemangiomas when they are found on endoscopy: establishing the diagnosis of cavernous hemangioma before an operation could save future patients from having a surgical intervention that may be unnecessary or larger than that required for this diagnosis. Additionally, by publishing this histologic finding, we hope that further studies will determine if this clue could be generalized to aid in diagnosis of cavernous hemangiomas from small biopsies in other organs or systems.

## 4. Conclusion

Small bowel cavernous hemangiomas are extremely rare vascular lesions which present with nonspecific GI symptoms such as abdominal pain and nausea, anemia, and GI bleeding. Complications include bowel obstruction, intussusception, perforation, and hemorrhage. Their size and propensity for bleeding may be affected by comorbid patient conditions which impair venous flow. Diagnostic challenges include nonspecific presentation and features on imaging, inconclusiveness of biopsy, or inaccessibility by endoscopy. Pathologists should be aware of the “endothelialized muscularis mucosae” which can be very helpful in diagnosing GI cavernous hemangiomas, particularly in small biopsies. The ability to acquire a histologic diagnosis preoperatively has important implications in patient counseling and surgical planning. A conservative surgical resection, which may be successfully performed laparoscopically, is recommended in order to relieve symptoms or prevent complications and provide definitive diagnosis when necessary.
